# miR-19a and miR-20a and Tissue Factor Expression in Activated Human Peripheral Blood Mononuclear Cells

**DOI:** 10.1155/2017/1076397

**Published:** 2017-10-30

**Authors:** Cristina Balia, Mirella Giordano, Valentina Scalise, Tommaso Neri, Gabriella Fontanini, Fulvio Basolo, Alessandro Celi, Roberto Pedrinelli

**Affiliations:** Dipartimento di Patologia Chirurgica, Medica, Molecolare e dell'Area Critica, Università di Pisa, Pisa, Italy

## Abstract

**Background and Aims:**

To investigate the behaviour of miR-19a and miR-20a, two microRNAs involved in posttranscriptional modulation of TF expression in peripheral blood mononuclear cells (PBMCs) exposed to high glucose (HG) and lipopolysaccharide (LPS), and to evaluate the involvement of angiotensin II in that process.

**Methods:**

TF Procoagulant Activity (PCA, one-stage clotting assay), antigen (Ag, ELISA), and miR-19a and miR-20a levels (specific TaqMan® MicroRNA Assays) were evaluated in PBMCs exposed to high glucose (HG, 50 mM), LPS (100 ng/mL), and Olmesartan (OLM, 10^−6^ M), an angiotensin II type 1 receptor antagonist.

**Results:**

HG increased TF expression and decreased both miRs as compared to control glucose conditions (11.1 mM). In HG-activated PBMCs, LPS stimulated TF expression and downregulated miR-20a, an effect reverted by OLM (10^−6^ M); miR-19a expression was unchanged by LPS in both CG and HG conditions.

**Conclusions:**

miR-19a and miR-20a are inhibited by inflammatory stimuli active on TF expression and their response differs by the stimulus under investigation; angiotensin II may participate in that mechanism.

## 1. Introduction

MicroRNAs (miRs) are small, ~22-nucleotide noncoding RNAs that inhibit transcriptional gene expression by interacting with sites of complementarity in the 3′ untranslated regions (3-UTR) of target mRNAs [e.g., [1]]. Posttranscriptional gene modulation by miRs involves several genes including Tissue Factor (TF) [2], the principal initiator of the clotting cascade, and a major regulator of haemostasis and thrombosis [3] expressed by circulating monocytes exposed to proinflammatory stimuli such as lipopolysaccharide (LPS, endotoxin) [3] and high glucose (HG) [4]. Locally generated angiotensin (AT)II, the effector arm of the renin angiotensin system (RAS), contributes actively to that inflammatory process [5], a mechanism downregulated by ATII type 1 receptor (AT1R) blockade [4, 5].

Among other potentially significant miRs interacting with TF gene [2], miR-19a and miR-20a have recently been shown to modulate TF expression in monocytes of patients with immune-mediated diseases [6]. That information, obtained in a very specific context, raises the issue of the behaviour of those two noncoding RNAs in response to stimuli active on TF expression in peripheral blood mononuclear cells (PBMCs) harvested from normal subjects activated by HG and LPS and whether ATII is involved in that relationship, an issue that has never been addressed insofar.

## 2. Materials and Methods

### 2.1. Cell Isolation and Culture

Human PBMC suspensions were obtained from unpooled buffy coats left over from blood bank draws taken from healthy donors, kept at room temperature, and utilized within a maximum of 4 hours from withdrawal. As detailed elsewhere [4], leukocytes were isolated from fresh buffy coats diluted 1 : 1 with sodium citrate 0.38% in saline solution, mixed gently with 0.5 volume of 2% Dextran T500, and left for 40 min for erythrocyte sedimentation. The leukocyte-rich supernatant was recovered and centrifuged for 10 min at 200 ×g. The pellet was resuspended in 30 mL of sodium citrate solution, layered over 15 mL of Ficoll-Histopaque, and centrifuged for 30 min at 400 ×g at 20°C. The PBMC-rich ring was recovered, washed twice in sodium citrate 0.38%, and resuspended in polypropylene tubes in RPMI 1640 medium supplemented with 100 U/mL penicillin-streptomycin.

Glucose perturbation was induced by supplementing PBMC cultures with D-glucose to reach a final concentration of 50 mM (from now on referred to as high glucose (HG)) to be compared with cells cultured in unsupplemented RPMI 1640 medium containing 11.1 mM D-glucose (from now on referred to as control glucose (CG)). Previous studies had excluded interferences derived from hypertonicity [4].

Drugs were kept in stock solution and diluted in serum-free RPMI at the appropriate concentrations immediately before use. Cell viability, as assessed by dimethyl thiazolyl diphenyl tetrazolium (MTT), was verified (>85% of viable cells) throughout all experimental phases.

The final PBMC preparations typically contain 25–35% monocytes, negligible proportions of neutrophils (<5%), and 65–75% lymphocytes.

All reagents and solutions used for cell isolation and culture were prepared with endotoxin-free water and glassware was rendered endotoxin-free by exposure to high temperature. Drugs were kept in stock solution and diluted in serum-free RPMI at the appropriate concentrations immediately before use.

Confounding from interindividual differences in TF sensitivity to proinflammatory stimuli was avoided by using unpooled buffy coats of the same subject throughout the different phases of the experimental series.

### 2.2. TF Procoagulant Activity (PCA)

PCA was assessed by one-stage clotting time in PBMCs disrupted by three freeze-thaw cycles as described in [4]. Time to clot formation was recorded and values converted to arbitrary units (AU) by comparison with a standard human brain TF calibration curve covering clotting times from 20 to 600 s. The standard TF preparation was arbitrarily assigned a value of 1000 AU/mL and a representative conversion of clotting times to AU is as follows: 100 AU-21 s, 10 AU-40 s, 1 AU-82 s, 0.1–187 s, 0.01 AU-375 s, and 0.001 AU- 600 s. Experiments were run in triplicate and averaged.

### 2.3. TF Antigen (Ag)

Cells were disrupted by three repeated freeze-thaw cycles and TF extracted with a buffer of Tris buffered saline (50 mM Tris, 100 mM NaCl, pH 7.4) containing 0.1% Triton X-100. After an overnight extraction, the debris was pelleted by centrifugation at 100 ×g for 1 h at 4°C and supernatants were used for ELISA (Imubind TF kit Sekisui Diagnostics, West Malling, United Kingdom). TF Ag levels were expressed in pg/mL using a reference curve created by the TF standards. Within and between assay variability were 3.5 and 5.5%, respectively.

### 2.4. miRs Expression

Total RNA including miRs was isolated from PBMCs using the miRNeasy Mini Kit (Qiagen), according to the manufacturer's instructions. RNA samples, after quality and quantity evaluation using a NanoDrop ND-1000 spectrophotometer, were stored at −80°C until used in the experiments. Quantification of miR-19a, miR-20a, and RNU6B (as housekeeping gene) expression was carried out in triplicate using specific TaqMan MicroRNA Assays (Applied Biosystems), according to the manufacturer's instructions. Briefly, 10 ng of RNA was retrotranscribed by the Taq-Man® MicroRNA Reverse Transcription (RT) Kit (Applied Biosystems) using individual miR-specific RT primers, and 1.3 *μ*l of RT product was analyzed by quantitative Real Time-PCR (qRT-PCR) on the Rotor-Gene 6000 (Corbett Research). Threshold cycle (Ct) and baselines were determined by manual settings. miR expression was calculated by relative quantification and fold expression changes were determined by the 2^−ΔΔCt^ method using the DataAssistTM software (Applied Biosystems). miR changes were analyzed as percent changes from either CG or HG used as referents.

TF PCA and Ag and miR levels were assayed after a 18 hr and 2 hr incubation interval, respectively. PCA, ELISA, and qRT-PCR results in both control and experimental groups were obtained from suspensions containing equal number of cells (3 × 10^6^ PBMCs/mL).

### 2.5. Experimental Design

The study included two series of experiments. The first series (*n* = 4) evaluated the effect on miR-19a and miR-20a and TF expression of HG versus CG and the second one (*n* = 9) evaluated the effect of LPS (100 ng/mL) stimulation on PBMCs incubated in CG or HG conditions, either per se or in presence of Olmesartan (OLM) (10^−6^ M), an ATII type 1 receptor (AT1R) antagonist [7].

### 2.6. Statistics

Statistical differences were tested by Wilcoxon test for paired comparisons on absolute data (TF PCA and Ag) or percent changes (miR expression) from control conditions normalized to 100%. Data were reported as means ± SD and a two-tailed *p*-level <0.05 was the threshold for statistical significance.

## 3. Results

**Figure 1:**
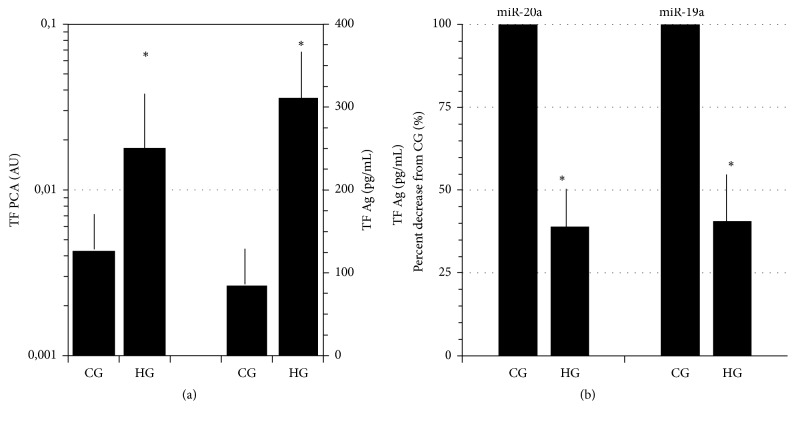
(a) High glucose (HG, 50 mM)-induced stimulation of TF PCA* (left ordinate, log scale)* and TF Ag* (right ordinate)*. (b) Downregulation of miR-20a and miR-19a expression in response to HG as compared with control glucose (CG, 11.1 mM). Means ± SD; *N* = 4, ^*∗*^*p* < 0.001 HG versus CG.

As compared with CG, HG inhibited miR-19a and miR-20a expression and induced a highly significant stimulation of both TF PCA and TF Ag (Figures 1(a) and 1(b)).

**Figure 2:**
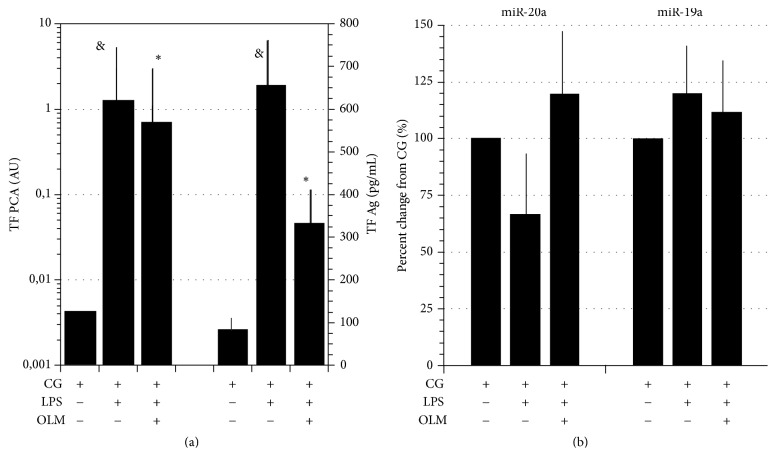
(a) LPS (100 ng/mL)-induced stimulation of TF PCA* (left ordinate, log scale)* and Ag* (right ordinate)* downregulated by AT1R blockade through Olmesartan (OLM, 10^−6^ M). PBMCs incubated in control glucose (CG, 11.1 mM). (b) Percent changes of miR-20a and miR-19a in LPS-stimulated PBMCs per se or in presence of OLM (10^−6^ M). Data expressed as percent changes from CG. Means ± SD, *N* = 9. ^&^*p* < 0.001 LPS versus CG; ^*∗*^*p* < 0.001 OLM + LPS versus LPS.

**Figure 3:**
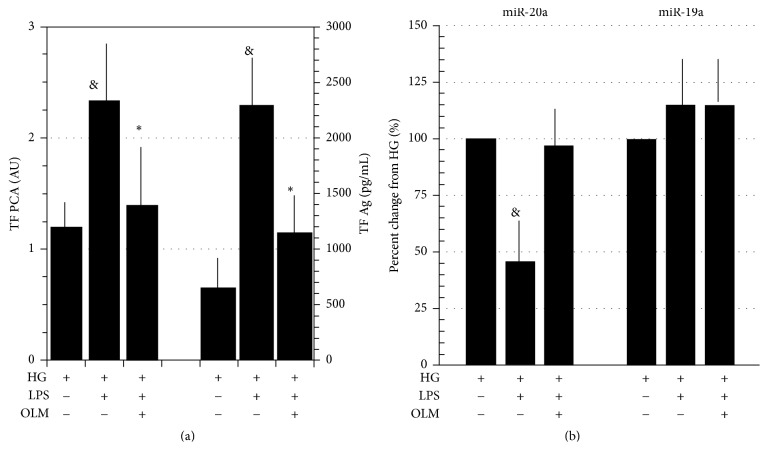
(a) LPS (100 ng/mL)-induced stimulation of TF PCA* (left ordinate)* (note the scale difference with the same parameter reported in Figure 2) and Ag* (right ordinate)* downregulation by AT1R blockade through Olmesartan (OLM, 10^−6^ M); PBMCs incubated in high glucose (HG, 50 mM). (b) Percent changes of miR-20a and miR-19a in LPS-stimulated PBMCs per se or in presence of OLM (10^−6^ M). Data expressed as percent changes from HG. Means ± SD, *N* = 9. ^&^*p* < 0.001 LPS versus HG; ^*∗*^*p* < 0.001 LPS + OLM versus LPS.

LPS increased TF PCA and TF Ag in both CG and HG conditions (Figures 2(a) and 3(a)). In CG conditions, miR-20a levels in response to LPS showed inconsistent changes but decreased in presence of HG, a trend reverted by AT1R blockade through OLM (Figures 2(b) and 3(b)).

miR-19a levels were insensitive to LPS stimulation in either of experimental conditions (Figures 2(b) and 3(b)).

## 4. Discussion

This study shows inhibition of both miR-19a and miR-20a in response to HG [4], a procoagulant [4, present results] and proinflammatory stimulus [8–10] that activates NF-*κ*B [10, 11], a redox-sensitive transcription factor [12] critical for TF gene expression [3]. Notably, cytoplasmic miR-induced silencing complexes restrain TF protein translation and destabilize TF mRNA by binding to the 3′-UTR of TF transcripts [1] and binding sites for both miR-19a and miR-20a have been recognized in the 3′-UTR of the TF mRNA transcript in human monocytes [6] and other cell types as well [13, 14]. Therefore, downregulated miRs expression by HG may conceivably facilitate HG-induced TF expression, a mechanism possibly contributing to the hypercoagulable state induced by high blood glucose in diabetic patients [15]. However, our PBMCs were perturbed by glucose concentrations far beyond that reached clinically and, therefore, the present results are to be seen as preliminary observations awaiting further validation in more physiological conditions. Moreover, we carried out these experiments in cell lysates, a preparation differing in several ways from intact cells such as, for example, amplification of procoagulant responses due to exposure of anionic phospholipids from disrupted membranes [16]. Therefore, the present results may not necessarily be extrapolated to intact PBMC, a point that deserves attention in future studies.

A second result of this study worth of comment relates to the inhibition by LPS of miR-20a in HG-activated PBMCs, an effect compatible with attenuation of a negative posttranscriptional feedback loop on TF expression in interaction with HG-induced excess ROS production and NFkB overactivation [8–11]. On the other hand, LPS stimulation did not change miR-19a levels in our PBMC preparations at variance, in this respect, with human umbilical vein endothelial cells (HUVECs), a cell line capable of rapid TF induction [17] in which endotoxin downregulated miR-19a expression [18]. Thus, the control mechanism of miR-19a may vary according to the tissue under evaluation, a speculative possibility in need of experimental verification.

A third and intriguing point emerging from these results was the reversal of LPS-induced miR-20a downregulation by OLM, an AT1R blocker that, as expected [4, 5], inhibited both TF PCA and TF Ag. That effect, reminiscent, to some extent, of results reported by other authors [19], suggests an involvement of ATII in posttranscriptional regulation of LPS-induced procoagulant activity. This speculative possibility adds a new hypothetical component to the intricate interrelationship between locally generated ATII and LPS [20–22], a major marker for the recognition of intruding Gram-negative bacteria by the innate immune system that initiates the pathogen-induced inflammatory response [23] of which activation of coagulation is a prominent component [24]. However, any role of ATII in the posttranscriptional control of inflammation- induced TF expression is only hypothetical at the moment and needs further studies including confirmation of the present findings by using other RAS antagonists such as ACE inhibitors and ATII receptor antagonists different from OLM.

## 5. Conclusions

The results of this study disclose behaviours of miR-19a and miR-20a potentially involved in the posttranscriptional regulation of TF expression in the context of the complex interplay among inflammation, coagulation, and ATII, in which TF plays a pivotal role.

## Abbreviations

Ag: Antigen
ATII: Angiotensin II
AT1R: Angiotensin II type 1 receptor
AU: Arbitrary units
CG: Control glucose
ELISA: Enzyme-linked immunosorbent assay
HG: High glucose
miR: MicroRNA
MTT: Dimethyl thiazolyl diphenyl tetrazolium
OLM: Olmesartan
PBMC: Peripheral blood mononuclear cell
PCA: Procoagulant activity
qRT-PCR: Quantitative real time polymerase chain reaction
RAS: Renin angiotensin system
RNA: Deoxyribonucleic acid
ROS: Reactive oxygen species
RPMI: Roswell Park Memorial Institute
SD: Standard deviation
TF: Tissue factor.

## Data Access

The datasets analyzed during the current study are available from the corresponding author on reasonable request.
